# Comparative transcriptomics and genomic analyses reveal differential gene expression related to *Colletotrichum brevisporum* resistance in papaya (*Carica papaya* L.)

**DOI:** 10.3389/fpls.2022.1038598

**Published:** 2022-12-23

**Authors:** Min Yang, Chenping Zhou, Hu Yang, Ruibin Kuang, Kaidong Liu, Bingxiong Huang, Yuerong Wei

**Affiliations:** ^1^ Key Laboratory of South Subtropical Fruit Biology and Genetic Resource Utilization (Ministry of Agriculture and Rural Affairs), Guangdong Province Key Laboratory of Tropical and Subtropical Fruit Tree Research, Institute of Fruit Tree Research, Guangdong Academy of Agricultural Sciences, Guangzhou, China; ^2^ Life Science and Technology School, Lingnan Normal University, Zhanjiang, China

**Keywords:** papaya, anthracnose, *Colletotrichum brevisporum*, DEGs, candidate genes

## Abstract

*Colletotrichum brevisporum* is an important causal pathogen of anthracnose that seriously affects the fruit quality and yield of papaya (*Carica papaya L.*). Although many genes and biological processes involved in anthracnose resistance have been reported in other species, the molecular mechanisms involved in the response or resistance to anthracnose in post-harvest papaya fruits remain unclear. In this study, we compared transcriptome changes in the post-harvest fruits of the anthracnose-susceptible papaya cultivar Y61 and the anthracnose-resistant cultivar G20 following *C. brevisporum* inoculation. More differentially expressed genes (DEGs) and differentially expressed long non-coding RNAs (DElnRNAs) were identified in G20 than in Y61, especially at 24 h post-inoculation (hpi), suggesting a prompt activation of defense responses in G20 in the first 24 h after *C. brevisporum* inoculation. These DEGs were mainly enriched in plant-pathogen interaction, phenylpropanoid biosynthesis/metabolism, and peroxisome and flavonoid biosynthesis pathways in both cultivars. However, in the first 24 hpi, the number of DEGs related to anthracnose resistance was greater in G20 than in Y61, and changes in their expression levels were faster in G20 than in Y61. We also identified a candidate anthracnose-resistant gene cluster, which consisted of 12 genes, 11 in G20 and Y61, in response to *C. brevisporum* inoculation. Moreover, 529 resistance gene analogs were identified in papaya genome, most of which responded to *C. brevisporum* inoculation and were genetically different between papaya cultivars and wild-type populations. The total expression dose of the resistance gene analogs may help papaya resist *C. brevisporum* infection. This study revealed the mechanisms underlying different anthracnose resistance between the anthracnose-resistant and anthracnose-susceptible cultivars based on gene expression, and identified some potential anthracnose resistance-related candidate genes/major regulatory factors. Our findings provided potential targets for developing novel genetic strategies to overcome anthracnose in papaya.

## Introduction

Papaya (*Carica papaya* L.) is a wildly cultivated tropical and subtropical fruit with a high nutritional and medicinal value (Chutichudet and Chutichudet, 2014; Ayón-Reyna et al., 2017; Yang et al., 2020). Consumer demand for papaya has been soaring due to the growing appreciation of its taste and nutritional value. The yield and quality of papaya are directly affected by infection of various pathogens, including *Colletotrichum* spp. (Bosquez-Molina et al., 2010; Ayón-Reyna et al., 2017; dos Passos Braga et al., 2019). *Colletotrichum* spp. can cause anthracnose, resulting in serious post-harvest loss of papaya fruits (dos Passos Braga et al., 2019). *Colletotrichum gloeosporioides* is commonly reported as the main causal agent of papaya anthracnose (Ali et al., 2015; dos Passos Braga et al., 2019). Recent studies have found that *Colletotrichum brevisporum* is also an important causal pathogen of anthracnose in papaya, which seriously affects fruit quality and yield (Vieira et al., 2013; Duan et al., 2018). The most effective and economical strategy for controlling papaya anthracnose is to mine resistance genes for molecular breeding resistant cultivars. However, studies on the control of papaya post-harvest anthracnose mainly focus on physical or chemical prevention (Zahid et al., 2012; Ali et al., 2016; dos Passos Braga et al., 2019). Few studies have explored the development of resistant cultivars and screened candidate resistance genes.

To defend against pathogens, host plants have developed various mechanisms to interact with pathogens. First, plants deploy a molecular surveillance system through cell surface-anchored pattern recognition receptors to detect pathogen- or microbial-associated molecular patterns and initiate pattern-triggered immunity (PTI) (Withers and Dong, 2017; Ramirez-Prado et al., 2018; Silva et al., 2018). PTI is effective against a broad range of microbes *via* inducing mitogen-activated protein kinases and/or calcium signals. These mitogen-activated protein kinases and/or calcium signals trigger a series of defensive responses to inhibit pathogen colonization (Schwessinger and Zipfel, 2008; Silva et al., 2018; Wang et al., 2018). However, adapted pathogens produce numerous virulence proteins called effectors to suppress or escape PTI and to achieve infection. In turn, host plants have developed a second line of receptors, known as intracellular nucleotide-binding site leucine-rich repeat (NBS-LRR) proteins. These receptors are activated by the specific recognition of cognate effector or pathogen avirulence (Avr) proteins, resulting in effect-triggered immunity (ETI) to inhibit pathogen growth (Thomma et al., 2011; Silva et al., 2018). Interestingly, ETI overlaps with PTI but induces stronger and longer-lasting responses compared to PTI.

In recent years, transcriptome sequencing has become essential for identifying genes involved in anthracnose resistance and exploring multiple underlying biological pathways. It has been successfully used to reveal the mechanisms and new candidate genes relevant to anthracnose resistance in many species, such as strawberry (Wang et al., 2017), tea (Wang et al., 2018; Shi et al., 2019), bean (Padder et al., 2016), maize (Schliebner et al., 2014), lentil (Bhadauria et al., 2017), and mango (Hong et al., 2016). These studies have identified multiple key anthracnose resistance-related pathways, such as plant-pathogen interactions, flavonoid biosynthesis, carbohydrate metabolism, and plant hormone signal transduction, and many anthracnose resistance candidate genes, including NBS-LRRs, pattern recognition receptors, mitogen-activated protein kinases, and transcription factors. Although anthracnose has seriously affected the quality and yield of post-harvest papaya fruits, very few studies have tried to identify the papaya’s molecular response and candidate resistance genes against *C. brevisporum* infection.

In this study, we compared and analyzed the transcriptome changes after *C. brevisporum* infection in two papaya cultivars (Y61 and G20) with different sensitivities to anthracnose, with the aim to identify potential genes/major regulatory factors associated with anthracnose resistance in papaya and to elucidate the response mechanism of post-harvest papaya fruits against *C. brevisporum* infection.

## Materials and methods

### Plant material, fungal isolates and pathogenicity tests

Papaya fruits with the same maturity level and without mechanical injury were selected from 2-year-old papaya trees of the anthracnose-susceptible Y61 and anthracnose-resistant G20 cultivars grown under standard field conditions at the Institute of Fruit Tree Research, Guangdong Academy of Agriculture Science, Guangzhou, Guangdong, China. The selected papaya fruits were washed with 75% alcohol and sterile water, air-dried, and used for *in vitro* pathogenicity tests. In addition to visual inspection, a colorimeter was used to measure the colour of the fruit peel. Following the CIE system, a, b, and L parameters were taken from six spots and then transformed in the hue angle, which was high if the peel was green and low if it was yellow (Fabi et al., 2007).

According to the isolation method described by Duan et al. (Duan et al., 2018), *C. brevisporum* was isolated from papaya fruits grown in common fields with anthracnose symptoms. For the needle-puncturing inoculation experiment, the papaya fruit was first punctured with a sterile needle. Then, 10μl spore suspension of *C. brevisporum* (10^8^ spores/mL) was inoculated at the wound site. Fruits inoculated with sterile water were used as the control. For the spray inoculation experiment, the spore suspension of *C. brevisporum* (10^8^ spores/mL) was directly sprayed on the surface of fruits. The sprayed fruits were then dried at room temperature and stored normally. The incidence of anthracnose in the fruit samples was observed and recorded every day. According to the anthracnose resistance classification criteria of papaya varieties described by He et al. (He et al., 2008), the anthracnose of papaya fruits was divided into nine grades: grade 0 was defined as no anthracnose spot; Grade 1 was defined as the spot area accounted for less than 5.0% of the fruit area and the diseased spot is small; Grade 3 was defined as lesions accounted for 6.0%~15.0% and the diameter of the spot increased to 1~2 cm. Grade 5 was defined as lesions accounted for 15.1%~25.0% and the lesions began to sink; Grade 7 was defined as lesions accounted for 25.1%~50.0% of the fruit area, and lesions gradually expanded into circular or irregular shape with brown flesh; Grade 9 was defined as the lesion area accounted for more than 50.0%, the flesh was rotten, and the lesion was stiff and easy to dig out. The calculation formula is as follows: the disease index =∑ (the number of fruits in each disease grade × disease grade) × 100/(the total number of fruits × the highest disease grade). The variety with a disease index of zero was defined as highly resistant (HR). The variety with a disease index between 0.1 and 10.0 was defined as R variety. The variety with a disease index between 10.1 and 30.0 was defined as moderately resistant (MR). The variety with a disease index between 30.1 and 40.0 was defined as moderately susceptible (MS). The variety with a disease index between 40.1 and 50.0 was defined as susceptible (S). The variety with a disease index greater than 50.1 was defined as a highly susceptible (HS) (**Table S1**). Each treatment consisted of three biological replicates, with 15 fruits per replicate.

### RNA-seq and data analysis

The fruits of Y61 and G20 were sampled at 0, 24, and 48 h after needle-puncturing inoculation. The samples were collected from fruit tissues within a diameter of 2 cm around the inoculation spot (excluding the inoculation spot). Each treatment consisted of three biological replicates, with five fruits per replicate. Eighteen samples were immediately frozen in liquid nitrogen and stored at –80°C, for RNA sequencing (RNA-seq).

Total RNA was isolated using the cetyltrimethylammonium bromide method described by Hao et al (Hao et al., 2017). DNA contamination was removed using RNase-free DNase I (TaKaRa Bio). RNA concentration was quantified using a NanoDrop™ 2000 System (Thermo Fisher Scientific, Wilmington, DE, USA) and RNA integrity was evaluated using an Agilent 2100 Bioanalyzer (Agilent Technologies, Santa Clara, CA, USA).

RNA-seq libraries were constructed on the Illumina NovaSeq 6000 Sequencing Platform using the Illumina TruSeq RNA Sample Prep Kit (Illumina Inc., San Diego, CA, USA). PolyA+ RNA isolation, fragment interruption, complementary DNA synthesis, adapter addition, polymerase chain reaction amplification, and RNA-seq were performed by Majorbio (Shanghai Majorbio Bio-pharm Technology Ltd., Shanghai, China).

The raw paired-end reads were trimmed, and their quality was controlled using fastp (Chen et al., 2018). The clean reads were then mapped onto the reference genome of Sunset papaya (Yue et al., 2022) using HISAT2 (Kim et al., 2019). Gene functions were annotated based on NCBI non-redundant protein sequences (NR; https://www.ncbi.nlm.nih.gov), Protein family (Pfam; http://pfam.xfam.org/), EggNOG (Clusters of Orthologous Groups of proteins; http://www.ncbi.nlm.nih.gov/COG/), Swiss-Prot (a manually annotated and reviewed protein sequence database, https://web.expasy.org/docs/swiss-prot_guideline.html), Kyoto Encyclopedia of Genes and Genomes (KEGG; http://www.genome.jp/kegg/), and Gene Ontology (GO; http://www.geneontology.org).

### Differential expression analysis and functional enrichment

DEseq2 (Love et al., 2014)was used to identify the differentially expressed genes (DEGs) between sample groups with a cut-off criteria of |log_2_ fold chang|≥1 and false discovery rate<0.05. HTSeq was performed to count the number of reads for each gene (Putri et al., 2022). Gene expression levels were calculated as fragments per kilobase of exon per million mapped reads. GO and KEGG enrichment analyses were performed by applying hypergeometric tests. A Bonferroni-corrected P-value ≤ 0.05 was used as the threshold for significant enrichment.

### Identification and analysis of long non-coding RNAs

To identify the lncRNAs in papaya, transcripts mapped to known RNA regions, such as mRNAs, ribosomal RNAs, transfer RNAs, small nucleolar RNAs, and small nuclear RNAs, were ignored. The remaining transcripts were considered if they were longer than 200 bp and contained more than two exons. Candidate lncRNA transcripts had to be supported by more than five reads. The coding potential calculator (CPC) (Kong et al., 2007) and coding potential assessment tool (CPAT) (Wang et al., 2013) were used to calculate the transcript encoding capabilities. Finally, the protein domains in the Hidden Markov Models library were searched for each candidate lncRNA transcript using pfamScan. Differential expression analysis of lncRNA was performed using the same method described above for differential expression analysis of mRNAs.

### Analysis of anthracnose resistance-related gene clusters

A previous study on common bean (*Phaseolus vulgaris*) identified a gene cluster containing 17 anthracnose resistance-associated genes located between 49512544 and 50023316 positions on chromosome 1 (Vaz Bisneta and Gonçalves-Vidigal, 2020). MCScanX was used to identify the synteny genes of this gene cluster between common bean and papaya (Wang et al., 2012). Genome annotation files in the GFF format and CDS files of papaya and common bean were used for the synteny analysis of their genomes. Synteny analysis was performed using the Python version of MCScanX (https://github.com/tanghaibao/jcvi). Jcvi was used to visualize the synteny of gene clusters between the two genomes.

### Domestication and resistance gene analogue analysis

RGAugury pipeline (Li et al., 2016) was used to identify RGAs in the papaya Sunset genome. RGAs include NBS-LRR, receptor-like kinase (RLK), receptor-like protein (RLP), and transmembrane coiled-coil (TMCC) domain protein candidate genes, which can be divided into 12 subfamilies.

We downloaded the VCF files of the single nucleotide polymorphisms (SNPs) (https://data.mendeley.com/datasets/m5phbmw43c/1) identified in the study by Yue et al. that re-sequenced 86 different varieties of papaya (Yue et al., 2022). SNP information of the flanking regions of the RGA genes were also extracted, ranging from 4 kb upstream to 4 kb downstream. The genetic distances (Fst) of RGAs among the solo, common, costa rican, and wild-type populations of papaya, and their flanking regions, were calculated using VCFtools v0.1.16 (Danecek et al., 2011). When calculating, the sliding window method was used, with 2.5 kb as the window and 50 bp as the step size. Expression correlations between transcription factors and RLPs were analysed using Pearson’s correlation test (false discovery rate ≤ 1e–6).

### Quantitative real-time PCR

To validate the RNA-Seq results, qRT-PCR assays were performed on the same RNA samples originally used for RNA sequencing. qRT-PCR was conducted on an Applied Biosystems 7500 Real-time PCR System using 2× SG Fast qPCR Master Mix (High Rox; TaKaRa) according to the manufacturer’s instructions. Six common DEGs of two cultivars were selected for qRT-PCR analysis after inoculation with C*. brevisporum*. Eukaryotic initiation factor 4A (EIF4A) amplification was used as the internal control (Zhu et al., 2012). Each examination was performed in triplicates with three biological replicates. Gene-specific primers were designed based on the coding sequences using Primer Premier 5.0 (**Supplementary Files 1**, **2**). The relative expression levels of each gene were calculated using the 2^-ΔΔCT^ method.

## Results

### Differential responses of G20 and Y61 to *C. brevisporum*

**Figure 1:**
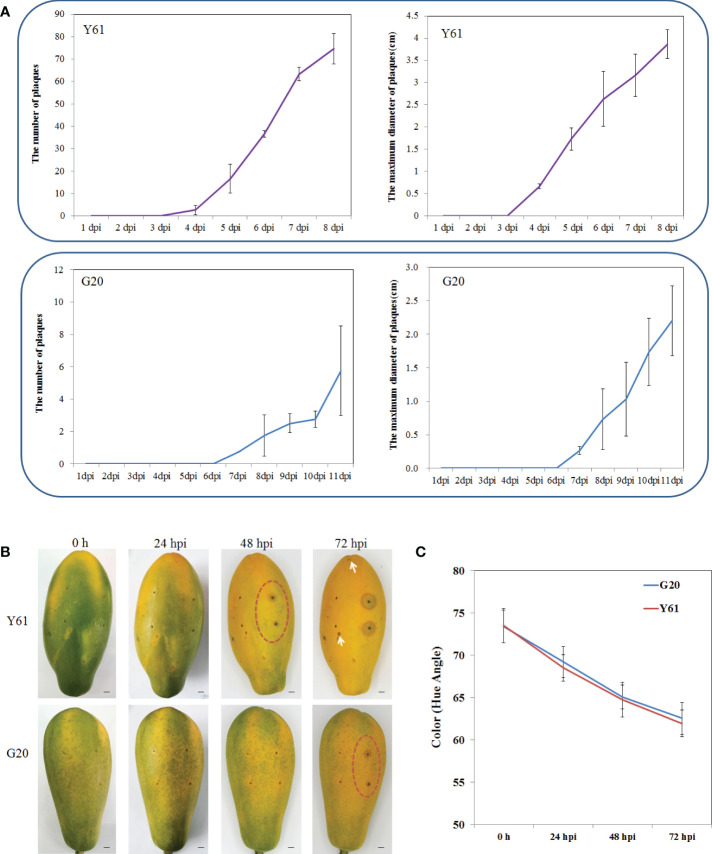
The difference of anthracnose tolerance between Y61 and G20 postharvest fruits after inoculated with *C. brevisporum in vitro*. **(A)** Statistical results of spray inoculation assay. Top, statistical results of the number and maximum diameter of anthracnose lesion plaques in Y61 from 1 to 8 dpi; bottom, statistical results of the number and maximum diameter of anthracnose lesion plaques in G20 from 1 to 11 dpi. **(B)** Results of needle puncturing inoculation assay. Each fruit was inoculated with four spots; two spots on the left were inoculated with sterile water as control, and the right was inoculated with *C. brevisporum*. The inoculation point in the red circle indicates the initial point of the anthracnose lesion plaque. The white arrow indicates the diffused lesion plaque outside the inoculation point in Y61. Bars=1cm. **(C)**. The fruits color development (angular hue) of Y61 and G20 at 0, 24, 48 and 72 hpi.

The G20 papaya cultivar showed significantly higher tolerance to anthracnose than the Y61 cultivar in the field, particularly in fruits. To confirm this result, we inoculated the post-harvest fruits of Y61 and G20 with conidial suspensions of *C. brevisporum in vitro* utilizing spray and needle-puncturing inoculation methods. In spray inoculation experiment, anthracnose lesions appeared 4 d post-inoculation in Y61 and 7 d post-inoculation in G20. After the onset of anthracnose, the number and size of lesions increased much slower in G20 than in Y61 (**Figure 1A**). According to the classification criteria of resistance to anthracnose of papaya varieties described by He et al. (He et al., 2008), G20 papaya had a disease index of 15.6, indicating it belongs to the middle resistance cultivars. Y61 papaya had a disease index of 60.0, suggesting it is a highly susceptible cultivar (**Table S1**). In the needle-puncturing inoculation experiment, papaya fruits with the same skin color and ripeness were inoculated with *C. brevisporum* (**Figures 1B, C**), typical brown anthracnose lesion plaques around the wounded areas appeared in Y61 fruits at 48 h post-incubation (hpi), but in the G20 fruit at 72 hpi. In Y61, brown anthracnose lesions around the wounded areas increased in size at 48 hpi, while the lesion plaques spread to other parts of the fruit at 72 hpi. However, in G20, lesion plaques of anthracnose formed only around the inoculation site, with no lesion plaques found in other parts of the fruit at 72 hpi (**Figure 1B**). These results confirmed that the G20 cultivar is more resistant to anthracnose than the Y61 cultivar.

### Identification of DEGs and DElncRNAs

**Figure 2:**
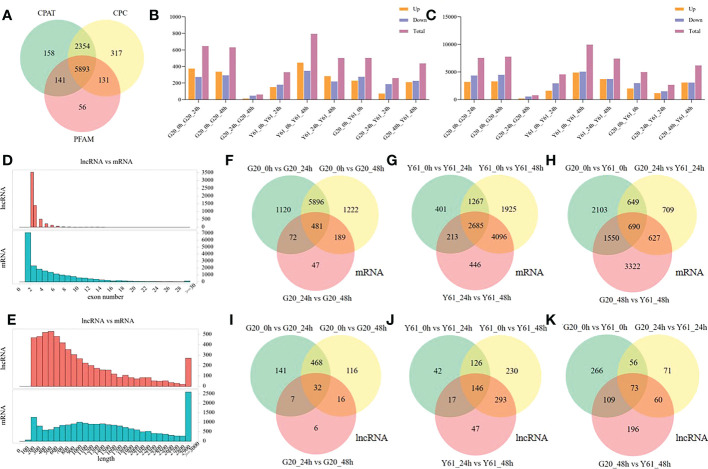
LncRNA identification and differential expression analysis of lncRNAs and mRNAs. **(A)** Venn diagram of the differentially expressed lncRNAs identified using CPAT, CPC, and PFAM in the 18 RNA-sequencing samples. **(B)** Number of up- and down-regulated lncRNAs between the different groups (Up is high expression in the former of the compared combination, while Down is low expression in the former of the compared combination); **(C)** Number of up- and down-regulated mRNAs between the different groups; **(D)** Comparison of the number of exons between the mRNA and lncRNA; **(E)** Comparison of the length distribution of the mRNA and lncRNA; **(F)** Venn diagram of the differentially expressed mRNAs between samples of G20 at different time points; **(G)** Venn diagram of the differentially expressed mRNAs between samples of Y61 at different time points; **(H)** Venn diagram of the differentially expressed mRNAs between samples of G20 and Y61 at a particular time point; **(I)** Venn diagram of the differentially expressed lncRNAs between the samples of G20 at different time points; **(J)** Venn diagram of the differentially expressed lncRNAs between samples of Y61 at different time points; **(K)** Venn diagram of the differentially expressed lncRNAs between samples of G20 and Y61 at the same time point.

The current reference genome for papaya has not been annotated for lncRNAs. In this study, 5983 lncRNAs were identified in 18 papaya RNA-seq samples (**Figure 2A**). Compared with lncRNAs, mRNAs have longer gene lengths and more exons (**Figures 2D, E**). The gene response patterns to *C. brevisporum* infection were inconsistent between the anthracnose-resistant cultivar G20 and anthracnose-susceptible cultivar Y61. Of these, Y61 had more genes that were differentially expressed, with 4901 genes expressed at high levels and 5072 genes expressed at low levels at 48 hpi, as compared to the expression levels at 0 hpi (**Figure 2C**). The G20 samples had 4499 genes expressed at high levels and 3289 genes expressed at low levels at 48 hpi, as compared to the expression levels at 0 hpi. At 24 hpi, more genes responded in G20 than in Y61, with 4365 high- and 3204 low-expression genes in G20 and 2958 high- and 1608 low-expression genes in Y61, as compared to those at 0 hpi. These results suggest that the genes in the susceptible cultivar Y61 were more likely to respond to long-term treatment. Interestingly, the patterns of DElncRNAs were similar to those of mRNAs, with the most DElncRNAs (403) identified in Y61, at 48 hpi (**Figure 2B**).

The response of the anthracnose-resistant cultivar G20 to anthracnose infection was similar at 24 hpi and 48 hpi, with similar numbers of DEGs at both time points compared with 0 hpi, and there were 6377 common genes were identified between both time points (**Figure 2F**). Moreover, the number of DEGs between these two time points was small, with a total of 789 DEGs, 481 of which were also differentially expressed between the 24 hpi vs. 0 hpi and 48 hpi vs 0 hpi conditions. In contrast, there was a higher number of DEGs between the 48 hpi vs 24 hpi conditions in Y61, *i.e.*, 7440, of which 2871 were the same as those differentially expressed between the 24 hpi vs 0 hpi conditions and 6781 were the same as those differentially expressed between the 48 hpi vs 0 hpi conditions (**Figure 2G**). There were 690 DEGs between G20 and Y61 at three time points (**Figure 2H**). The DElncRNAs between G20 and Y61 were also time specific, with only 73 DElncRNAs occurring in all three periods (**Figure 2K**). 500 DElncRNAs were identified between the 24 hpi vs 0 hpi and 48 hpi vs 0 hpi conditions in G20 (**Figure 2I**), and 272 DElncRNAs were identified between the 24 hpi vs 0 hpi and 48 hpi vs 0 hpi conditions in Y61 (**Figure 2J**). These results indicate that the patterns of genes and lncRNAs responding to *C. brevisporum* infection differ significantly between cultivars, with the response time of the genes of resistant cultivars being shorter than that of the susceptible cultivars.

### Analysis of gene clusters associated with anthracnose resistance

**Figure 3:**
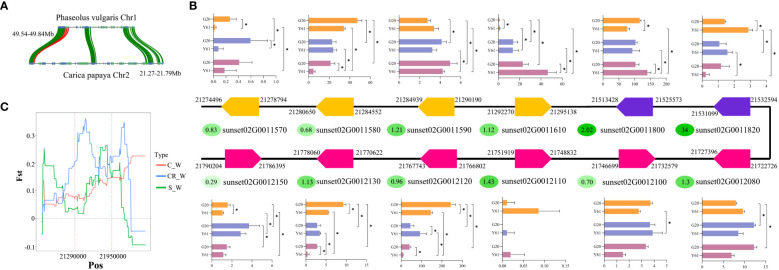
Analysis of gene clusters associated with anthracnose resistance. **(A)** Synteny of gene clusters associated with anthracnose resistance in common bean and papaya genomes. **(B)** Schematic representation of the arrangement of genes in a candidate gene cluster for anthracnose resistance in papaya. Arrows to the right indicate that the gene is in the forward orientation on the chromosome, while arrows to the left indicate that it is in the reverse orientation. The number in the oval next to each gene is the Ka/Ks value for that gene in the papaya population. The bar graphs indicate the expression level of a gene in different groups, with an asterisk indicating whether the gene is significantly differentially expressed between the two groups. The 3 different colours from the top to the bottom of the bar graph indicate 0, 24, and 48 hpi, respectively. **(C)** Fst values of the sunset02G0011610 gene in the gene cluster between different cultivar populations and the wild-type population. S indicates solo, CR indicates costa rican, C indicates common, and W indicates wild-type.

A previous study on common bean (*Phaseolus vulgaris*) identified a gene cluster of 17 resistance genes associated with anthracnose resistance. In the current study, genomic synteny analysis led to the identification of a gene cluster (**Figure 3A**) homologous to the cluster identified in common bean within chromosome 2 of papaya, 21.27–21.96 M, which had 12 resistance genes (**Figure 3B**). Among them, five genes (sunset02G0011580, sunset02G0011610, sunset02G0011800, sunset02G0012120, and sunset02G0012130) were differentially expressed between G20 and Y61 following *C. brevisporum* infection. In this study, the ka/ks values of the resistance genes within the gene clusters were calculated from the SNPs identified in 86 papaya genomes in a study by Yue et al. (Yue et al., 2022). It was found that three of these five genes, which were differentially expressed between the cultivars, were subject to positive selection. However, their ka/ks values were not particularly higher than 1. In contrast, sunset02G0011580 had a ka/ks value of 0.68, indicating that it was subjected to purifying selection. Sunset02G0011820 was under the most positive selection pressure and was downregulated under both the 48 hpi vs 0 hpi and 48 hpi vs 24 hpi conditions in Y61. It suggested that this gene was subjected to positive selection pressure during the domestication of papaya, resulting in a large variation in the genotype of this gene within different cultivars. The variations may have led to a change in the gene expression in response to pathogen infection. Fst analysis showed that the gene region of sunset02G0011610 was genetically divergent between the costa rican and wild-type populations (**Figure 3C**).

### Genome-wide identification and analysis of RGAs

**Figure 4:**
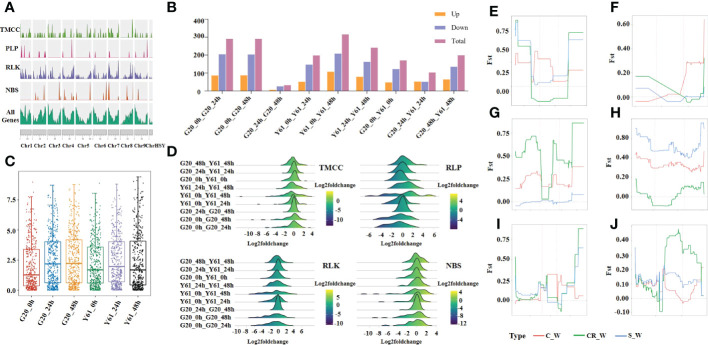
Genome-wide identification and analysis of RGAs in papaya. **(A)**, Density map of chromosome distribution of different RGA types on papaya chromosomes. **(B)**, Number of differentially expressed RGA genes between groups; **(C)**, Box-plot of RGA gene expression (FPKM) in different groups; **(D)**, log_2_(fold-change) distribution; **(E–J)**, Fst values between sunset02G0021170, sunset06G0008770, sunset07G0001330, sunset07G0007900, sunset07G0021830, and sunset09G0019560 in costa rican (CR), common (C), and solo (S) populations and wild-type (W) population.

In the present study, 529 RGA genes were identified based on the Sunset papaya reference genome, of which 105 were TMCC, 53 were NBS-LRR, 348 were RLK, and 23 were RLP (**Figure 4A**). The different resistance genes were not evenly distributed on the chromosomes. For example, there were 20 TMCC genes on chr7 while six on chr8. Moreover, 422 RGAs were differentially expressed between different time points for the same cultivar or between different cultivars at the same time point (**Figure 4B**). The median expression of RGAs in G20 was lower than that in Y61 at 0 hpi but still higher than that in Y61 at 24hpi and 48 hpi (**Figure 4C**). These results suggest that the increase in the total expression dose of the RGA gene helped papaya resist *C. brevisporum* infection. In the *C. brevisporum*-infected samples, the number of significantly upregulated RGAs was fewer in G20 than in Y61. RGAs highly expressed in G20 was less than that highly expressed in Y61, and the peak log2(fold-change) between G20 and Y61 was showed in **Figure 4D**. We selected six differentially expressed RGAs and found that they had genetic differences in the 4 kb upstream and downstream regions of the gene or in the gene itself (**Figures 4E–J**). For example, sunset07G0007900 displayed large genetic differences in the 4 kb upstream and downstream regions of the gene as well as in the gene between the solo and wild-type populations. In contrast, there were fewer differences between the costa rican and wild-type populations. These results suggest that these RGA genes had been subject to divergent selection pressures when the wild-type species were domesticated into different cultivars.

**Figure 5:**
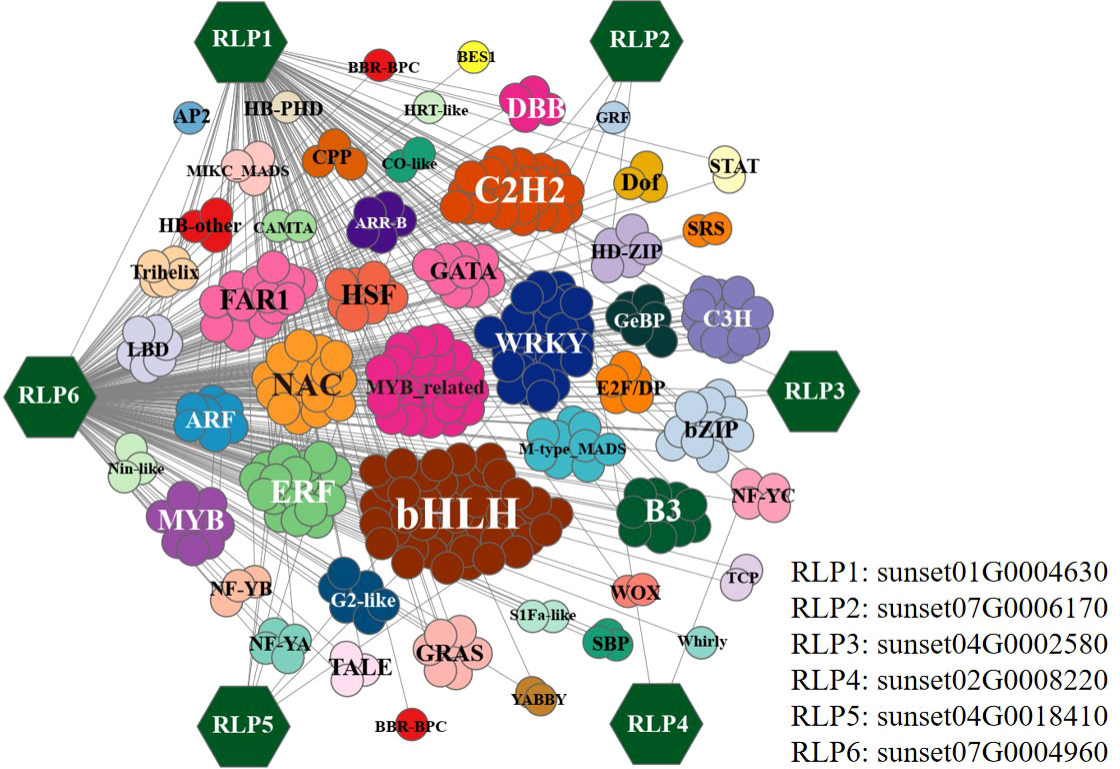
Co-expression network of differentially expressed RLP genes with the involved transcription factors. The colour-filled hexagons represent the RLP genes. Correlations between TFs and RLP genes were calculated using a Pearson’s correlation test (P ≤ 1e–6).

This study investigated the regulation of differentially expressed RGA genes by various transcription factors. For example, correlation coefficients between differentially expressed transcription factors and differentially expressed RLP genes were calculated, and a large number of genes involved in regulating RLP were identified, based on a threshold of P ≤ 1e–6. Among them, the transcription factors bHLH, C2H2, and WRKY were identified as the most important ones involved in the regulation of the differentially expressed RLP genes (**Figure 5**). For example, sunset07G0006170, sunset04G0002580, and sunset02G0008220 are regulated by a few transcription factors, whereas sunset01G0004630 and sunset07G0004960 are regulated by many transcription factors. These results suggested that transcription factors can regulate RGA genes, but they are specific to resistance genes and not all RGA genes are regulated by the same transcription factors in response to *C. brevisporum* infection.

### GO enrichment analysis of DEGs and target genes of DElncRNAs

**Figure 6:**
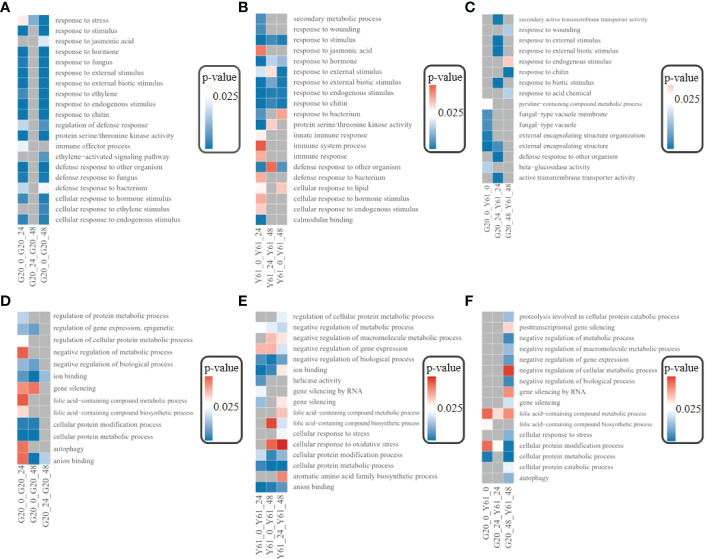
Results of GO enrichment analysis of DEGs. **(A)**, GO terms for the enrichment of DEGs in G20; **(B)**, GO terms for the enrichment of DEGs in Y61; **(C)**, GO terms for the enrichment of DEGs between G20 and Y61 at different time points; **(D)**, GO terms for the enrichment of target genes for DElncRNAs in G20; **(E)**, GO terms for the enrichment of target genes for DElncRNAs in Y61; **(F)**, GO terms for the enrichment of target genes for DElncRNAs between G20 and Y61 at different time points.

DEGs between G20 and Y61 after *C. brevisporum* infection were enriched in GO terms, such as response to stimulus, response to external stimulus, and response to hormones (**Figures 6A, B**), suggesting that many genes are functionally similar between the resistant and susceptible papaya cultivars in response to *C. brevisporum* infection. However, some DEGs may function differently in anthracnose-resistant and -susceptible papaya cultivars. For example, the DEGs involved in the “response to fungus” GO term were significantly enriched in G20 but not in Y61, suggesting that more genes responding to fungal infection are involved in response to *C. brevisporum* infection in G20 but not in Y61. In G20, many GO terms were not enriched in the DEGs between the 24 hpi vs 48 hpi conditions. However, in Y61, the DEGs between the 24 hpi vs 48 hpi conditions were enriched in several GO terms associated with plant stress response, suggesting that the anthracnose-resistant cultivar G20 responds to *C. brevisporum* infection at as early as 24 hpi. In contrast, the susceptible cultivar Y61 responds to *C. brevisporum* infection much later. A large number of DEGs between G20 and Y61 were also enriched in pathways such as fungal-type vacuoles and fungal-type vacuole membranes (**Figure 6C**), further indicating the differences between the two cultivars in response to *C. brevisporum* infection.

The GO terms enriched in the target genes of DElncRNAs were different from those enriched with the DEGs (**Figures 6D, E**), such as autophagy and gene silencing, indicating that the target genes of DElncRNAs did not coincide with most DEGs. DElncRNAs in G20 started responding at 24 hpi, so there were only a small number of enriched GO terms for the target genes of DElncRNAs between the 24 hpi vs 48 hpi conditions. However, many DElncRNAs in Y61 also responded after 48 hpi. The target genes of the DElncRNAs between Y61 and G20 were enriched in many GO terms at 48 hpi, such as negative regulation of gene expression, negative regulation of cellular metabolic processes, and cellular responses to stress (**Figure 6F**).

### Effects of *C. brevisporum* infection on the expression of genes involved in flavonoid and phenylpropanoid biosynthesis

**Figure 7:**
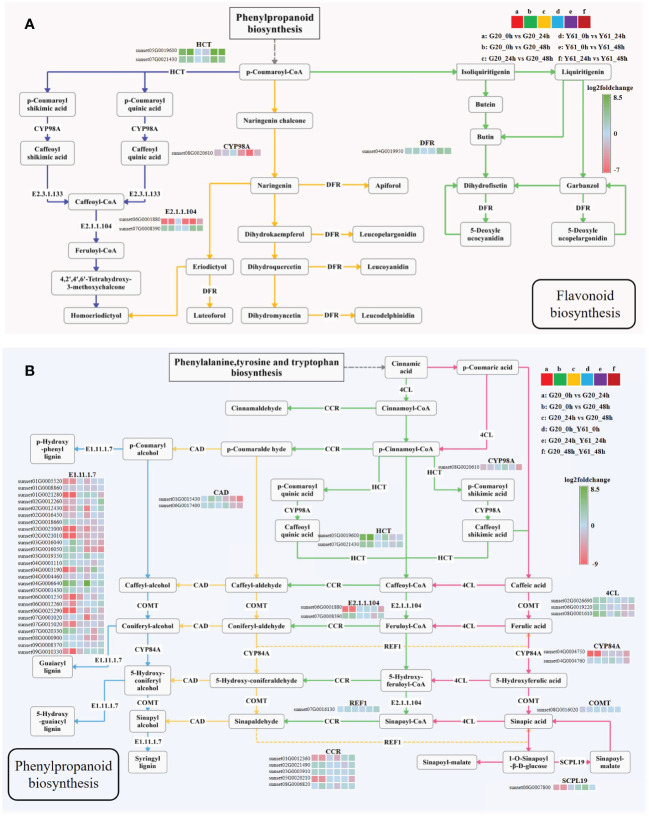
The expression patterns of differentially expressed genes in the flavonoid **(A)** and phenylpropanoid biosynthesis **(B)** pathways. The fold-change is the ratio of the former group to the latter group. The enzymes involved are: shikimate O-hydroxycinnamoyltransferase (HCT), 5-O-(4-coumaroyl)-D-quinate 3’-monooxygenase (CYP98A), caffeoyl-CoA O-methyltransferase (E2.1.1.104), bifunctional dihydroflavonol 4-reductase/flavanone 4-reductase (DFR), peroxidase (E1.11.1.7), cinnamyl-alcohol dehydrogenase (CAD), coniferyl-aldehyde dehydrogenase (REF1), cinnamoyl-CoA reductase (CCR), 4-coumarate-CoA ligase (4CL), ferulate-5-hydroxylase (CYP84A), caffeic acid 3-O-methyltransferase/acetylserotonin O-methyltransferase (COMT), and serine carboxypeptidase-like 19 (SCPL19).

KEGG pathway enrichment analysis revealed that the DEGs of G20 after *C. brevisporum* infection were enriched in the flavonoid biosynthesis pathway (**Figure 7A**). In addition, DEGs of G20 and DEGs between the two cultivars were enriched in the phenylpropanoid biosynthesis pathway after *C. brevisporum* infection (**Figure 7B**). The results showed that the expression of HCT and DFR in the flavonoid biosynthesis pathway was downregulated after *C. brevisporum* infection. In Y61, HCT and DFR showed a continuously decreasing pattern of expression at 24 hpi and 48 hpi, because the expression of HCT and DFR was still significantly different between the 24 hpi vs 48 hpi conditions in Y61. The expression pattern of genes in the pathway was inconsistent. For example, CYP98A gene was not differentially expressed in G20 but upregulated in Y61. Caffeoyl-CoA O-methyltransferase (E2.1.1.104) gene has two homologs with different expression patterns, which might be the reason for their functional differentiation.

The homologous genes encoding peroxidase (E1.11.1.7) in the phenylpropanoid biosynthesis pathway were partially differentially expressed between G20 and Y61, and showed a greater fold-change in G20. Among them, sunset01G0021280, sunset02G0021000, sunset02G0021010, sunset09G0010330, and sunset06G0025290 were expressed at higher levels in the control samples of G20 than in the samples treated for 24 hpi and 48 hpi. Many other DEGs in this pathway showed greater fold-changes within G20 than between the two varieties, such as ferulate-5-hydroxylase (CYP84A), cinnamoyl-CoA reductase (CCR), and HCT. These results suggested that the DEGs involved in flavonoid and phenylpropanoid biosynthesis have different expression patterns in different cultivars, which may influence the accumulation of these secondary metabolites and the response to *C. brevisporum* infection.

### Plant-pathogen interaction pathway is important in the response of papaya to *C. brevisporum* infection

**Figure 8:**
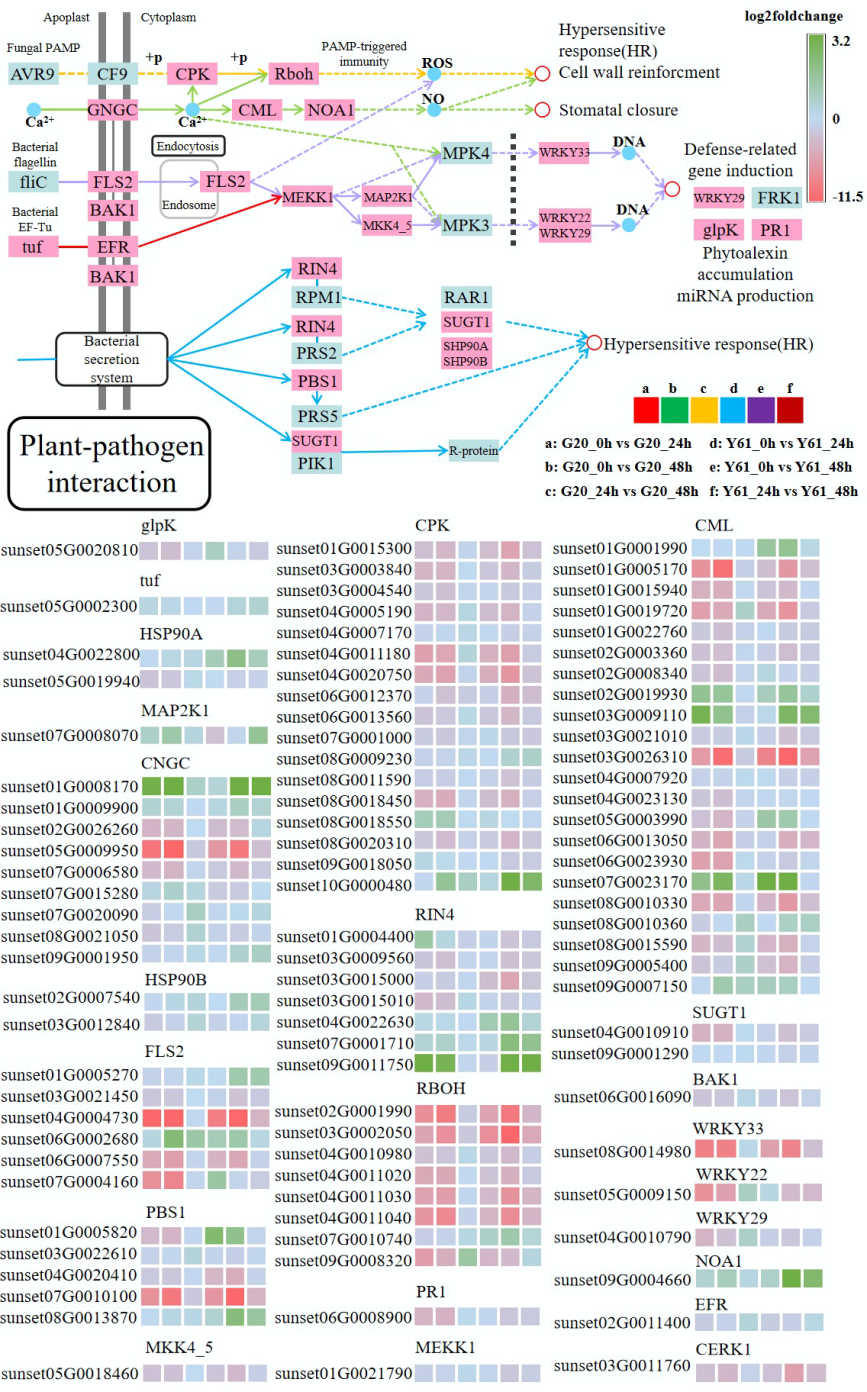
Differentially expressed genes in the plant-pathogen interaction pathway. The fold-change is the ratio of the former group to the latter group. The enzymes involved are: glycerol kinase (glpK), elongation factor Tu (tuf), molecular chaperone HtpG (HSP90A), mitogen-activated protein kinase kinase 1 (MAP2K1), cyclic nucleotide-gated channel, plant (CNGC), heat shock protein 90 kDa beta (HSP90B), suppressor of G2 allele of SKP1 (SUGT1), calcium-dependent protein kinase (CPK), mitogen-activated protein kinase kinase 4/5 (MKK4_5), mitogen-activated protein kinase kinase kinase 1 (MEKK1), brassinosteroid insensitive 1-associated receptor kinase 1 (BAK1), LRR receptor-like serine/threonine-protein kinase (FLS2), WRKY transcription factor 33 (WRKY33), WRKY transcription factor 22 (WRKY22), WRKY transcription factor 29 (WRKY29), nitric-oxide synthase, plant (NOA1), LRR receptor-like serine/threonine-protein kinase (EFR), serine/threonine-protein kinase (PBS1), respiratory burst oxidase (RBOH), calcium-binding protein (CML), pathogenesis-related protein 1 (PR1), and RPM1-interacting protein 4 (RIN4).

The plant-pathogen interaction pathway (map04626) is a summary of the response patterns of plants to pathogen infection. Therefore, the response patterns of the two papaya cultivars to *C. brevisporum* infection in this classical response pathway were examined, even without including many unknown responses. As shown in **Figure 8**, most genes involved in this pathway showed very similar expression patterns in Y61 and G20, suggesting that the response patterns are similar in the resistant and susceptible cultivars. Most genes were upregulated after infection. Many genes in this pathway have homologs, such as calcium-dependent protein kinase (CPK) and calcium-binding protein (CML). Because of sequence variation, some homologous genes may have diverse functions, resulting in different expression patterns. For example, sunset03G0026310 was expressed at high levels, sunset07G0023170 was expressed at low levels after infection. Moreover, the expression patterns of some homologous genes were inconsistent between G20 and Y61. For example, sunset07G0010740 (RBOH) was downregulated with a high fold-change in Y61 at 48 hpi, while this did not occur in G20.

### Validation of RNA-Seq data by quantitative real-time PCR analysis

**Figure 9:**
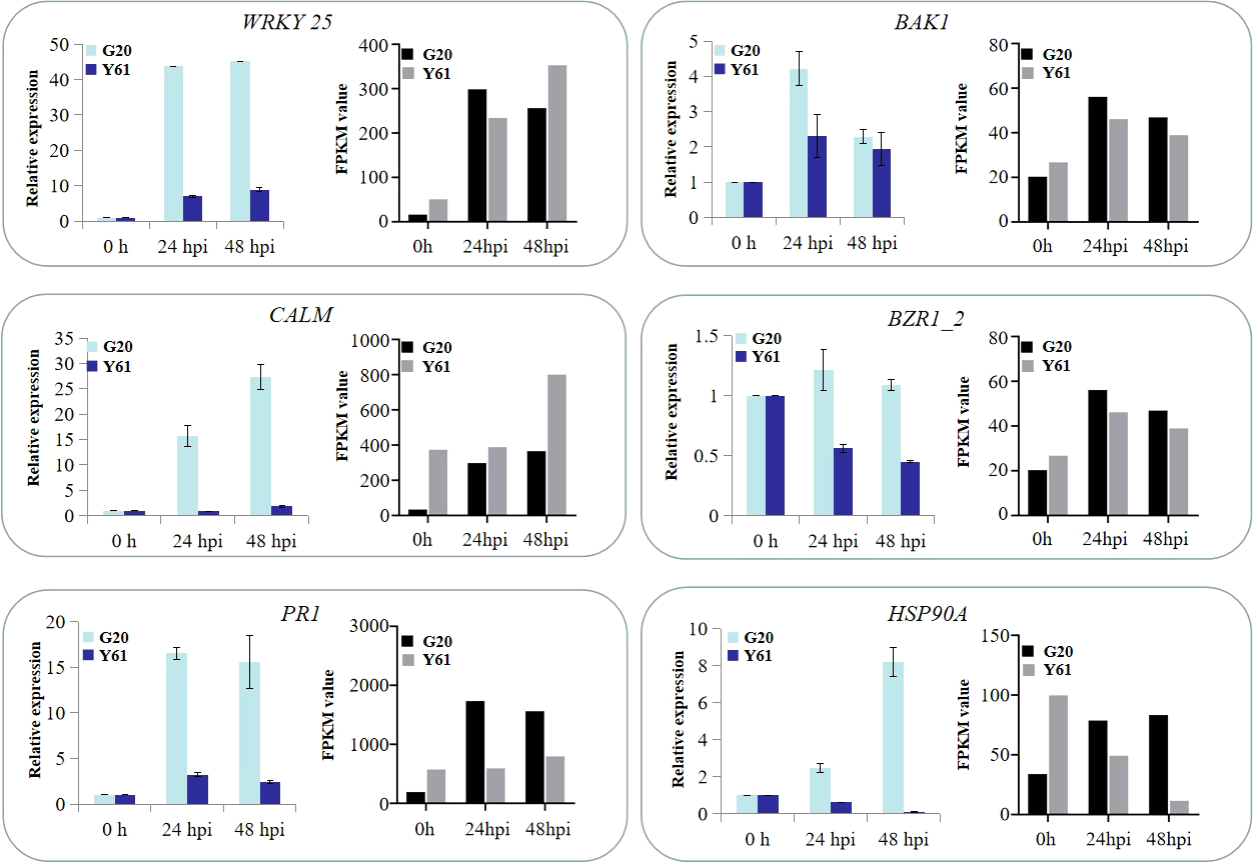
Expression profile comparisons between RNA-Seq and qRT-PCR data. The relative expression levels of six DEGs after *C. brevisporum* infection, including WRKY transcription factor 25 (WRKY 25), brassinosteroid insensitive 1-associated receptor kinase 1 (BAK1), CALM, brassinazole resistant 1-like (BZR1_2), pathogenesis-related protein 1-like (PR1), and heat shock protein 90 (Hsp90A).

To verify the RNA-Seq results, six DEGs (WRKY 25, BAK1, CALM, BZR1_2, PR1 and Hsp90A) from RNA-Seq data were randomly selected for verification by qRT-PCR. The results showed that the expression patterns of these DEGs were similar between qRT-PCR and RNA-Seq data at different times after inoculation (**Figure 9**), indicating that the RNA-Seq data were reliable.

## Discussion

Anthracnose causes severe losses in papaya production (Madani et al., 2014). However, little is known about the molecular responses and candidate resistant genes in post-harvest papaya fruit after *C. brevisporum* infection. The results of pathogenicity tests in this study clearly showed that cultivar G20 is more resistant to anthracnose than cultivar Y61. The transcriptome changes between the two cultivars after *C. brevisporum* infection using RNA-seq identified that more genes in G20 started responding at 24 hpi. In comparison, more genes in Y61 started responding at 48 hpi. The time-specificity of gene expression during growth and development or in response to disease infection is a common phenomenon in animals and plants (Sun et al., 2018; Bai et al., 2022). In plants, lncRNAs regulate the expression of their target genes at the transcriptional level and play essential roles in response to infection by various pathogenic microorganisms (Sharma et al., 2022). However, the role of lncRNAs in response to biotic stress in papaya has not been reported. This study found that lncRNAs have a similar response pattern to mRNAs, with lncRNAs in the resistant cultivar G20 responding shortly after infection. The present study identified target genes of DElncRNAs involved in many pathways, some of which are related to plant response to pathogen infection. For example, autophagy, a conserved degradation and recycling process in eukaryotes, controls stress adaptation and programmed cell death and is an important regulator of plant innate immunity (Zhou et al., 2014; Hofius et al., 2017). This study found that the target genes of the DElncRNAs between G20 and Y61 at 48 h after *C. brevisporum* infection were enriched in the GO term of autophagy, which provides a novel underlying mechanism for understanding the differences in disease resistance in papaya.

In plants, gene clusters play an important role in the synthesis of secondary metabolites and response to disease infection (Darma et al., 2019; Jeon et al., 2020), and resistance genes are also often present as gene clusters. Several studies have reported that resistance gene clusters function in resistance to pathogen infection (Barragan and Weigel, 2021; Chovelon et al., 2021). Anthracnose is a serious disease affecting plant growth and development. A study on common bean identified a gene cluster (Vaz Bisneta and Gonçalves-Vidigal, 2020) that confers resistance to anthracnose. This is a valuable reference for our research, so we performed a comparative genomic analysis of papaya and common beans. This study identified a gene cluster in the papaya genome based on synteny analysis of the papaya and common bean genomes. Most genes in this cluster were responsive to *C. brevisporum* infection, indicating that it is also essential in papaya. In particular, sunset02G0011610 not only responded to *C. brevisporum* infection at the expression level but also displayed genetic differences between various cultivar populations and wild-type populations, thereby providing a basis for mining genotypes with greater resistance to the disease.

RGAs are important genes in plants that respond to biotic stress. Their genotypes are important for plant resistance to diseases. Wild-type papaya is generally more resistant than cultivars to a broader range of pathogens (Dillon et al., 2006; Sengupta et al., 2014; Tineo et al., 2020). We found that some differentially expressed RGA genes significantly differed in the fixation index (Fst) between the cultivars and wild-type populations. For example, the gene region and 4 kb upstream and downstream regions of sunset07G0021830 had the largest Fst differences between the solo and wild-type populations. In contrast, these differences were small between the costa rican and wild-type populations. In the 4 kb upstream region of another gene, sunset07G0007900, there was a large Fst difference between the costa rican cultivar and the wild-type, but a smaller Fst difference in the gene region. These results suggest that some genetic differences arise in different RGAs due to different growing environments when forming different cultivars from the wild-type species. Genetic differences arise in genes, and the *cis*-regulatory regions of RGAs, such as the 4 kb upstream region. Such differences in RGA genes between cultivars and wild populations likely contribute to papaya disease resistance. The genetic differences in the RGA genes may result in richer allele and more abundant haplotypes . Different haplotypes potentially allow for phenotype variations across varieties (Zhang et al., 2021). Our findings suggest that further evaluation of haplotypes in RGA genes in multiple varieties and assessment of disease resistance in these varieties may be an effective way to identify disease resistance genes.

RGA genes showed a significant transcriptional response in both resistant and susceptible cultivars, suggesting that they play an important role in disease resistance regardless of cultivars’ disease resistance status. In contrast, the differentially expressed RGA genes between G20 and Y61 were not highly expressed in G20. Instead, 48 h after *C. brevisporum* infection, more RGAs were highly expressed in Y61. However, as shown in **Figure 4C**, the overall expression of RGA genes was higher in G20 than in Y61 at 48 h after *C. brevisporum* infection, suggesting that RGA expression level may contribute to the greater resistance of G20. These overall RGA expression levels may be influenced by several factors, such as the regulation of methylation and transcription factors (Liu et al., 2015; Tirnaz et al., 2020). Many transcription factors identified in this study were potential regulators of RGAs.

The accumulation of phenylpropanoid and flavonoid metabolites is important in plant responses to biotic and abiotic stresses. A study found that the accumulation of phenylpropanoid and flavonoid metabolites contributes to pathogen and drought resistance in apples (Geng et al., 2020). The DEGs of G20 identified in this study were enriched in the flavonoid synthesis pathway, suggesting that the accumulation of these two metabolites is also important for papaya response to *C. brevisporum* infection. In plants, *HCT* gene is involved in the biosynthesis pathways of flavonoids and other metabolites and plant resistance to adverse environments (Wang and Balint-Kurti, 2016; Zhang et al., 2018). Our study showed that *HCT* gene was downregulated after *C. brevisporum* infection in G20, indicating that fewer products are produced through it in the pathway, and that the upstream products are mainly catalysed by other enzymes, such as the CYP98A, in response to disease infection. The phenylpropanoid biosynthesis pathway was enriched not only in DEGs of G20 but also in DEGs between G20 and Y61. However, it was not enriched in DEGs of Y61, suggesting that this pathway plays a unique role in G20. Peroxidase (E1.11.1.7) is not only involved in the phenylpropanoid biosynthesis pathway, but also a component of the antioxidant defence system (Mir et al., 2015). The peroxidase gene has several family members, some of which were upregulated while others were downregulated after infection. Further exploration of specific gene family members from their expression patterns would also be useful for papaya breeding. *SCPL19* gene in the phenylpropanoid biosynthesis pathway was found located in a quantitative trait locus for resistance to *Puccinia striiformis* in wheat, suggesting that *SCPL19* gene may play an important role in plant responses to fungal infection (Rollar et al., 2021). We found that *SCPL19* gene was upregulated in G20 after *C. brevisporum* infection and had higher expression levels in G20 than in Y61 at 24 hpi.

Peroxisomes are present in all eukaryotic cells and play important roles in plant growth, development, and response to various stresses (Su et al., 2019). In this study, DEGs in G20 and Y61 were enriched in the peroxisome pathway (**Supplementary Figure 1**). Some genes in the peroxisome pathway had similar expression patterns between the two cultivars, whereas others had different expression patterns. For example, sunset03G0015500 (acetyl-CoA acyltransferase 1) was upregulated in both cultivars at 24 hpi. Sunset03G0015500 was also upregulated in *Nerium indicum L.*, in response to witches’ broom disease (Wang et al., 2022). Another gene, sunset05G0005410 (EPHX2), showed a higher fold-change of downregulation in Y61 after *C. brevisporum* infection. The differential expression patterns of these different members in one gene family warrant further exploration of possible candidate genes. The rich homology of CML and CPK in the plant-pathogen interaction pathway is a valuable resource for further research. Some steps in the plant-pathogen interaction pathway are catalyzed by one gene, such as WRKY22 and PR1, which play important roles in plant pathogen infection (Breen et al., 2017; Long et al., 2021).

In summary, this study explained the mechanism of different sensitivities of G20 and Y61 to anthracnose based on gene expression and screened potential candidate genes/major regulators associated with anthracnose resistance. Our findings will help develop novel genetic strategies to overcome anthracnose in papaya.

## Data availability statement

The original contributions presented in the study are publicly available. This data can be found here: NCBI Sequencing Read Archive (SRA), PRJNA692338.

## Author contributions

MY and YW perceived and planned the study, MY performed most of the experiments and all of bioinformatic analysis. CZ, HY, RK, KL and BH helped collected the samples and extracted total RNAs for qPCR. MY wrote the manuscript. All authors contributed to the article and approved the submitted version.

## Supplementary material

Supplementary file 1 Primers used for qRT-PCR in this study.

Supplementary file 2 The coding sequence (CDS) of six DEGs.

Supplementary Figure 1 Differentially expressed genes in the peroxisome pathway. The fold-change is the ratio of the former group to the latter group. The enzymes invovled are: isocitrate dehydrogenase (IDH1), xanthine dehydrogenase/oxidase (XDH), acyl-CoA oxidase (ACOX1), sarcosine oxidase/L-pipecolate oxidase (PIPOX), phytanoyl-CoA hydroxylase (PHYH), alanine-glyoxylate transaminase/serine-glyoxylate transaminase/serine-pyruvate transaminase (AGXT), malonyl-CoA decarboxylase (MLYCD), hydroxymethylglutaryl-CoA lyase (HMGCL), long-chain acyl-CoA synthetase (ACSL), NAD+ diphosphatase (NUDC), superoxide dismutase, Fe-Mn family (SOD2), superoxide dismutase, Cu-Zn family (SOD1), peroxin-2 (PEX2), acetyl-CoA acyltransferase 1 (ACAA1), soluble epoxide hydrolase/lipid-phosphate phosphatase (EPHX2), dehydrogenase/reductase SDR family member 4 (DHRS4), (S)-2-hydroxy-acid oxidase (HAO), acyl-CoA thioesterase 8 (ACOT8), 2-hydroxyacyl-CoA lyase (HACL1), 2,4-dienoyl-CoA reductase [(3E)-enoyl-CoA-producing], peroxisomal (DECR2), peroxin-3 (PEX3), peroxin-19 (PEX19), peroxin-1 (PEX1), peroxin-6 (PEX6), peroxin-7 (PEX7), peroxin-5 (PEX5), peroxin-12 (PEX12), peroxin-13 (PEX13), peroxin-14 (PEX14), peroxisomal membrane protein 2 (PXMP2), protein Mpv17 (MPV17), Mpv17-like protein (MPV17L), solute carrier family 25 (peroxisomal adenine nucleotide transporter), member 17 (SLC25A17), and alcohol-forming fatty acyl-CoA reductase (FAR).

Supplementary Table 1 Identification results of resistance to anthracnose in two papaya cultivars Y61 and G20.
