# Supplementary material for: Comparative transcriptomics and genomic analyses reveal differential gene expression related to Colletotrichum brevisporum resistance in papaya (Carica papaya L.)
Source: Front Plant Sci. 2022 Dec 23;13:1038598. doi: 10.3389/fpls.2022.1038598 (PMC9816866; doi:10.3389/fpls.2022.1038598)
Supplement: Supplementary file 2 — The coding sequence (CDS) of six DEGs. [file DataSheet_2.docx]

>WRKY25

ATGGCGTCTTCCTACGGCGGTAGACTTGATACATCGACAAACTTACACCAGATAACTACAGCTGGGAGCAGCTTCACTTTCCCTACCCACTCATTCATGA

ACACATCTTTCACTGGCCTCCTCAACTCCTCTTCTCTCGACGACGATGAACAGCTCAAGGTCGCCGCTGTAAATTCCGCTGATGAAACCACCAGGCGTGG

TGGGTTATCGTTGTCGGATCGAATCGCGGAGAGAACCGGGTCGGGTGTGCCCAAGTTTAAGTCGATATCGCCTCCTTCTCTCCCTCTCTCTCCTCCTCCT

TTCTCGCCTTCTTCTTACTTTGCTATTCCTCCTGGGTTGAGCCCAGCTGAGCTTTTGGATTCTCCTGTACTGTTAAGCTCTTCTAATATTTTGCCGTCCC

CAACAACTGGAACTTTTCCAGCTCAAGCTTTCAATTGGAAGGGCAATAACCCACAAGAAATCAAACAAGAAAACACAAACAATTACTCTGATTTCTCTTT

TCAGCCCCGACCAAGACCACCCACATCATCAACTGGCTTCCAATCTTCTGCAACAACAACAACAATCACAACTGCAACACAACAGTCATGGAGTTTCCAA

GAATCTTCCAAGCAAGATATGGTAAAACCCGATTATTCTTCCATGCAGAGCTTCTCTCCTGAGATTTCTACCATACAGACTACCACCCAAAGCACCAATA

AGACCTCTGGGTTCCAATCGGATTGTGGGAATTACCATCAACAACAGCCACAGGGTCACAGAGAAAGCAGGAGATCAGATGATGGGTATAATTGGAGAAA

ATATGGGCAGAAACAAGTGAAAGGAAGTGAAAATCCAAGGAGTTATTACAAGTGCACATACCCAAATTGTCCAACCAAGAAGAAAGTTGAAAGGTCTTTA

GATGGGCAGATTACTGAGATCGTTTATAAGGGTACCCATAACCATCCTAAGCCTCAGTCTACTAGGAGATCATCATCATCATCATCCTCATCGTCCATAG

CTTCTTCTCATGTAATTCAGGGCTCTAATCAACTTTCTGGTGAAATCCCAGATCAGTCATTTGTCACAAATGGTATTGGTCAGATGGATTCTGCTGCGAC

ACCAGATAATTCTTCCATATCAATAGGGGATGATGACTTTGATAGGAGCTCCCAGAAGAGTAAATCTGGTGGTGATGATCTTGATGAAGATGAACCTGGT

GCCAAAAGATGGAAAATAGAAGGGGAAAATGAAGGAATTTCAGCTCCTGGGAGCAGAACTGTGAGGGAACCTAGAGTTGTGGTCCAAACAACCAGTGACA

TAGATATTCTAGATGATGGATACAGATGGAGGAAATATGGGCAGAAAGTAGTCAAGGGAAACCCAAATCCAAGGAGTTACTATAAGTGCACATACCCTGG

TTGCCCTGTAAGAAAGCATGTTGAGAGGGCATCCCATGATCTTAGGGCAGTGATCACCACCTACGAGGGAAAACACAACCATGACGTCCCACCTGCTCGT

GGCAGCGGAAGCCATTCTATTAATAGGCCACCATTTCCTGATAACAACAACAATAGCAATAGCAACAATGCAGCAATGACAATAAGGCCGATAGCTAATG

TCACCGACAACTCTATAACTTATCCTCTCCGCAACTTAAGGCAGCAATCATCCGAAGGGCAATCACCCTTAAGCCTAGACATGCTGCAGAGCCCTGGGAG

TTTCGGGTTCCCGGGATTCGGAAACCCTTTGGGGTCTTACATCCCTCAGGCACCAAACACCAATAATATGTTCCCTAGAGCAAAGGAAGAACCAAGAGAT

GACTTGTTTCTTGAAGCGCGCGGCGGCTGGAAGAGCAGGAAGGAAGTTGACGGAGGGGGGGAGGAAGAAGAAGAGAGAGAGACCGAAAGTGAAAGGAACC

GAGAATTAAGTGGTGGAGGGAGTGAAATAAAAAAAAAAGGGGGCTTTTTTTTGTGGATTTTTGGGTTGAGAAGAAGGGAGAACCCGAGCGTGAGAAGAGA

AGGATCCAACCGAAGAAGGGTGTGTAATTACCCTGAGGATTTAACTCTAACACTGTATGCTACAAGTGAGGATTTGAAGCAAGGAAGAAGGAATACCTTT

GTACATTTTGTTCGGTTTTTCTTAATTCTTTTCCTGTCCCTGTCATTTGCAATAATGTCGGTCACGGCAGGTGTTAGTGATACTATAATAGCAATTAGGG

ATAAGCTAAGAGGTAAAATTGGGCAGACGAAAGTAAAGAGGTACTGGCCTGGTAAAGCTCCTGAATGGGCTGATGAGGGTGATGAAGATGGGGATATTAG

GATGCAGAGGGCAGCTGCTGTGGAGAGAGCTTTCCCTAGTCAGGAAGATTCAGGTGTTGTCAGGAAAGATGATCGAAGGCTGCGTCGTTTGGCTGAGAGC

AGAATAGACAACCGTGAAGAAGTGAGAGCTGATCACCGGCGCATTCGACAAGCTGAGATTGTTTCAACAGAAGAAGAGGAAAACAGAAGACAAGAGGGAC

TGGATGCGGAGGAAGAGGATGAGAATGCATTGGAAGAAAGAAGAAGAAGAATTAGGGAAAAGATGCTTCAAAGGGAACAAGAAGAGGCTGCCCTCCTTCC

AGAAGAAGAGGAGGAGGAGCCAGAAGAGGAGGAAGAGGAATCTGAGTATGAGACTGATTCAGAAGAAGAACTGCCAGGTATTGCAATGGTCAAGCCTATC

TTTGTGCCGAAAGCTGAGAGAGAGACTATTGCTGAACGTGAGCGACTTGAAGCTGAAGAGCGGGCCATTGAGGAATTAGCCAAGAGGAAGCTAGAGGAGA

GAAAAGTAGAGACGAAGCAAATAGTTGTTGAAGAGATACGGAAAGATGAAGAGATTCGCAAGAATATGGAATTGGAGGCAAATGTTGCTGATGTGGATAC

TGATGATGAACTTAACGAGGCAGAGGAGTATGAAGCTTGGAAAGCAAGAGAGATTGCACGGATCAAGAGGGATAGGGAGGACCGTGAGGCAATGTTAAAG

GCAAAGGAAGAGATTGAGAAGGTAAGGAACATGACTGAGGAAGAAAGGAGGGAATGGGAGAGGAAGAATCCAAAGCCTGTTCCCCCATCAAAGCAGAAGT

GGAGGTTTATGCAGAAATACTACCACAAGGGTGCTTTCTTCCAATCAGATGCTGATGACCTTGCTGGAACTGTTGGATCAGATGGTATATACCAGCGTGA

TTTCTCTGCTCCTACCGGAGAAGATAAGATGGACAAGACTATATTGCCAAAGGTCATGCAGGTCAAACACTTTGGTCGTAGTGGGAGGACAAAATGGACT

CATCTCGTCAATGAGGATACAACTGATTGGAACAATCCATGGACGTATAATGATCCTCTTAGGGCTAAGTATAATGCAAAGATGGCGGGAATCAATGCAC

CCATAGCAAAACCCAAAGGAAGCAAGAAGTTGAAGGATTGGGAGACCTGA

>PR1

ATGATGAAGGTTAAAGTTTCAGTAGCAGTAGCAATTGCTTTGATCTCGTGCATGGTGTTAGGCAGCCATGCACAGGACTCCCCCCAAGATTTCCTGAGCG

CCCATAACGCAGCTCGTGCAGCCGTCAGCGCTGCTCTTCCGCCTCTCACTTGGGACACCACTGTCGCCGGCATCGCGCAGAACTACGCCAACCAACGCAA

AGCTGACTGCGCTTTGCGACACTCCGGTGGTCCTTACGGCGAGAACATTGCATGGGGCAGCGCCAACTTGTCCGGCATGGACGCTGTCAACATGTGGGTT

GCGGAGAAAGCTAATTATGATTATAATTCAAATACATGCGCTCCTGGAAAAGTTTGTGGGCATTATACTCAAGTTGTGTGGAAAAACTCGGTTCGTTTAG

GGTGTGCAAAAGTGACGTGCAACAATGGTGGAACTTTCATCACTTGCAACTATGATCCTCCAGGCAACTTTATTGGCCAGAAGCCTTACTAA

>CALM

ATGTGTCCGTCTGACAGAATCCTCCGGCCCGCAGCGGCTGCCAGGTCCGATTTCAGATCCGCCTTCGACGTGATCGACGCCGATCACGATGGGAAGATCA

GCGGCGATGATCTCCGTATGTTCTACGTCGGCTATTACGGTGGAGGATCGGGAGACGTTGATGACTTGATCGGGACGATGATGAAGGTTGCTGACTCTAA

TAAGAACGGATTCGTCGAGTACGATGAGTTCGAGAGCGTTTTGAAGGGGCATAATGGAATTAGTTGCGGTGGCGGTGGCGGATTAATGGAGGATATGTTC

AAGGTGATGGATAAGGATGGCGATGGCCGGTTGAGCCACCAGGATTTGAAGAGTTACATGGAGTTGGCTGGTTTTCCGGCTAGTGATGAAGATATCTCGG

CTATGATCAGATTGGCTGGCGGTGATGACAAAGAAGGAGTCTCCCTTGACGGCCTGCTCAAGGTTCTTTCATTTTCATCTTTACTCTGA

>BAK1

ATGATGCAGCGAATGATGTCGGCTTTTTTGTGGTTAGTTTTGGTGTTCGATTTAGCTATGAGAGTCGCTGGTAATGTAGAAGGTGATGCATTGAATTCTT

TGAAGTCCAAGTTAAATGACCCTGACAATGTGCTACAAAGTTGGGATGCTACCCTTGTCAATCCATGTACATGGTTTCATGTTACATGCAGTGCTGAAAA

TAGTGTAACACGAGTTGATCTTGGAAATGCAAATCTATCTGGTCAACTGGTTCCAGACCTTGGGCGGCTTCAAAATTTGCAGTATTTGGAACTTTATAGC

AATAACATAATTGGAGAAATTCCAGAGGAGCTTGGCAATTTGTCCGAGTTGGTCAGCTTGGATCTTTACTTGAACAAGTTAAATGGTCATATCCCTCCAA

CTTTGGGAAAGCTTACAAAACTACGCTTCTTGCGTCTCAATAACAACACGTTATCGGGCATTATTCCTGGGTCTTTGACTACTGTTACATCCCTACAAGT

CCTGGATCTCTCAAACAATAATCTCACAGGAGATATTCCTGTCAATGGTTCTTTTTCACTATTTACTCCTATCAGTTTTGCAAACAATAAACTCAATGCT

CTTCCAGCTTCCCCACCTCCTCCTATCTCACCTACTCCATCTTCTTCAGGTAGCAGTACAACTGGAGCTATTGCTGGGGGAGTTGCCGCAGGTGCTGCAT

TGCTGTTTGCTGCCCCTGCTATTGCTCTTGCTTGGTGGCGAAGAAGGAAACCGCAAGATCATTTCTTTGATGTACCTGCTGAAGAGGACCCAGAAGTTCA

TTTGGGGCAACTCAAGAGGTTTTCTCTGCGTGAACTGCAAGTTGCAACAGATAATTTTAGCAACAGAAATATTCTGGGTAGAGGTGGATTCGGCAAGGTT

TACAAAGGACGCCTAGCTGATGGTTCTCTGGTTGCAGTGAAAAGATTGAAAGAGGAGCGTACTCAAGGTGGAGAGCTGCAGTTCCAAACAGAGGTAGAAA

TGATCAGCATGGCAGTTCATCGGAATCTGCTTCGTCTACGTGGCTTTTGCATGACCCCTACAGAGCGGTTGCTAGTCTACCCCTTTATGGTTAATGGAAG

TGTAGCATCATGTTTAAGAGAACATCCAGAATCACAACCACCGCTCAGTTGGCCAATACGGAAGCGAATTGCATTGGGATCTGCAAGGGGGCTTGCGTAT

TTGCATGATCATTGTGACCCAAAGATTATTCATCGTGATGTGAAAGCTGCAAACATATTGTTAGATGAAGAATTTGAAGCTGTTGTTGGAGATTTCGGGC

TTGCTAAACTCATGGACTACAAAGACACCCATGTAACCACAGCAGTACGTGGAACAATTGGTCATATAGCTCCTGAGTACCTCTCAACTGGAAAATCATC

TGAGAAAACTGATGTTTTTGGGTATGGGGTCATGCTTCTTGAACTGATCACGGGACAGAGGGCTTTTGATCTTGCTCGGCTTGCTAATGATGATGATGTG

ATGTTGCTTGATTGGGTAAAAGGACTACTGAAAGATAAGAAATTGGAAACACTAGTTGATCATGATCTGCAAGGTAATTATGATGAACATGAGGTGGAGC

AACTCATCCAAGTGGCTCTGTTGTGTACTCAAGGCTCTCCGATGGAACGACCCAAGATGTCAGAGGTGGTCAGAATGCTGGAAGGTGATGGGTTGGCCGA

AAGATGGGAAGAATGGCAGAAGGAGGAGATGTTCCGGCAAGAATTTAACCATACTCACCACCATCCAAGTACGGCTTGGATTGTTGCTGACTCGACTTCT

CATATTGCTGCAGACGAATTATCTGGTCCTAGATGA

>BZR1_2

ATGACATCCGACGGTGCAACCTCCACGTCGGCGGCGCCTCCCAGGAGGAAGCCGTCCTGGAGGGAGAGAGAGAACAATAGGAGGAGGGAGAGGAGGAGGA

GAGCCATAGCCGCCAAGATATACGCTGGCCTTAGAGCTCAAGGTAATTATAATTTGCCTAAGCACTGCGATAACAACGAGGTCTTGAAGGCTCTATGTTC

CGAGGCTGGTTGGATCGTTGAAGAAGATGGTACTACCTACCGCATGGGATGCAAGCGACACCCAAATGATATAGCGGGAACTTCCGCTAAAATCACTCCA

TACTCTTCCCAAAATCCAAGTCCACTCTCCTCATCATTTCCAAGTCCAATTCCTTCCTACCAAGTAAGTCCTTCTTCCTCTTCCTTCCCCAGCCCCACTA

GAATCGATCCCAGCACCTCTTCCACAATCCTTCCTTTCCTTCGGAATGCAATTCCTTCATCTCTACCACCTCTTCGAATCTCCAATAGTGCCCCTGTGAC

TCCACCATTATCCTCCCCAACCTCAAGAAATCCCAAGCCAGTGCCCAACTGGGACTCCATTGCCAAGGAGTCCATGGCCTCCTTCAATTACCCTTTCTAT

GTGGTGTCTGCCCCAGCTAGCCCAACCCACCGCCAATTTCACACCCCGGCCACCATACCTGAATGTGATGAATCCGATACTTCAACTGTTGATTCTGGTC

AATGGATAAGCTTTCAGAAATTTGCCCCTTCTGTCTCTGCGGTGCCTACCTCCCCAACCTTCAATCTTGTGAAACCAGTGGCTCCGCAAATGTCCCCCAA

TGGTTTGATCAATGAGGCAGGACGGAGTTCGGAGTTCCAGTTTGAGAATGGACAGGTGAAGCCATGGGAAGGGGAGAGGATCCATGAAGTGGGATTAGAC

GATCTAGAGCTTACCCTTGGAAATGGAAAGGTTCGGAGTTGA

>HSP90A

ATGGCGGATGTGCAGATGGCTGATGCAGAGACCTTCGCCTTTCAGGCCGAGATTAACCAGCTTCTGAGTTTGATTATCAACACATTTTACAGCAACAAGG

AGATTTTCCTTCGTGAGATTATCAGCAACTCATCTGATGCTCTTGACAAGATTCGATTCGAGAGCTTAACTGACAAGAGCAAGCTCGATGCTCAACCAGA

GCTCTTCATTAGGCTCGTTCCTGACAAGGTCAACAAGACCCTCTCCATCATAGACAGCGGTATCGGCATGACCAAAGCAGATTTGGTCAACAATTTGGGT

ACCATTGCTAGGTCAGGAACGAAGGAGTTCATGGAGGCCTTGCAGGCTGGAGCTGACGTGAGCATGATTGGTCAATTTGGTGTTGGTTTCTACTCAGCTT

ACCTTGTTGCAGAGAAGGTGATCGTAACAACAAAGCACAATGATGACGAGCAATACATCTGGGAATCCCAAGCTGGCGGTTCCTTCACAGTTACCAGGGA

TGTCAATGGTGAGCAACTTGGCAGGGGAACGAAGATCACTCTCTTCCTCAAGGAGGACCAGCTGGAGTACCTGGAGGAGAGAAGGATTAAGGACCTTGTG

AAGAAGCATTCTGAGTTCATCAGCTACCCCATCTATCTCTGGACTGAGAAGACAACCGAGAAGGAAATTAGTGATGATGAGGATGATGAACCCAAAAAGG

AAGAGGAAGGAGATATTGAGGAGGTTGACGAGGAAAAAGAGACCAAATCAAAGAAGAAGAAGATCAAGGAGGTTTCTCATGAGTGGCAACTCATCAACAA

GCAGAAGCCTATCTGGTTGCGCAAGCCCGAGGAGATCACCAAGGAGGAATATGCCTCCTTCTACAAGAGCTTGACCAATGACTGGGAGGATCACCTTGCC

GTGAAACACTTCTCTGTCGAGGGTCAACTTGAGTTCAAAGCGATTCTATTTGTACCGAAAAGGGCGCCATTTGATCTTTTCGACAGCAGGAAGAAGATGA

ACAATATCAAACTCTATGTCCGCAGAGTCTTTATCATGGACAACTGTGAGGAACTCATCCCCGAGTTCCTCGGATTCGTTAAGGGTGTCGTTGACTCTGA

TGACTTACCACTCAACATCTCTCGTGAGATGCTTCAGCAGAACAAGATTCTGAAGGTAATTAGAAAGAACCTTGTTAAAAAATGCATTGAAATGTTCAAT

GAGATTGCAGAGAACAAAGAAGACTACAACAAGTTCTATGAGGCATTCTCGAAGAACCTGAAATTGGGCATCCATGAGGATAGCCAGAACAGAGCGAAAC

TGGCAGACCTCCTTCGCTACCACTCAACCAAGAGTGGAGACGAATGGACAAGCTTGAAGGACTATGTTACCAGAATGAAGGAGGGACAAAAAGATATTTA

CTACATTACAGGTGAGAGCAAGAAAGCCGTTGAGAACTCACCATTCTTGGAGCGACTCAAAAAGAAGGGATACGAAGTGCTCTTCATGGTGGACGCCATT

GATGAGTATGCTGTCGGCCAACTAAAGGAATATGACGGGAAGAAGCTCGTCTCCGCTACCAAGGAAGGTCTGAAGCTGGACGATGAAACAGAGGAAGAGA

GAAAGAAAAAGGAAGAGAAGAAAAAATCATTCGAGGACCTGTGCAAAGTAATCAAGGACATATTAGGAGACAAGGTGGAGAAGGTGGTAGTGTCCGACAG

AATTGTGGATTCTCCTTGCTGTTTGGTAACCGGAGAGTACGGGTGGACAGCCAACATGGAGAGAATCATGAAAGCTCAGGCCCTTAGAGACAGCAGCATG

AGTTCATACATGTCGAGCAAGAAGACAATGGAAATCAACCCTGACAACGGCATCATGGAGGAGCTCAGGAAGAGGGCTGAAGCTGACAAGAACGACAAGT

CTGTCAAAGATCTTGTCTTGCTGCTCTTTGAGACTGCTCTCCTGACTTCTGGTTTCAGCCTTGATGATCCAAACACATTTGCTTCTAGGATTCACAGAAT

GCTGAAGTTGGGTCTGAGCATTGATGAGGAGGAGACTGCTGGTGATGATGATGACATGCCGCCTCTGGAGGAAGATGGCGCTGAGGAGAGCAAGATGGAG

GAGGTCGATTAA
