# Supplementary figures and images for: Comparative transcriptomics and genomic analyses reveal differential gene expression related to Colletotrichum brevisporum resistance in papaya (Carica papaya L.)
Source: Front Plant Sci. 2022 Dec 23;13:1038598. doi: 10.3389/fpls.2022.1038598 (PMC9816866; doi:10.3389/fpls.2022.1038598)

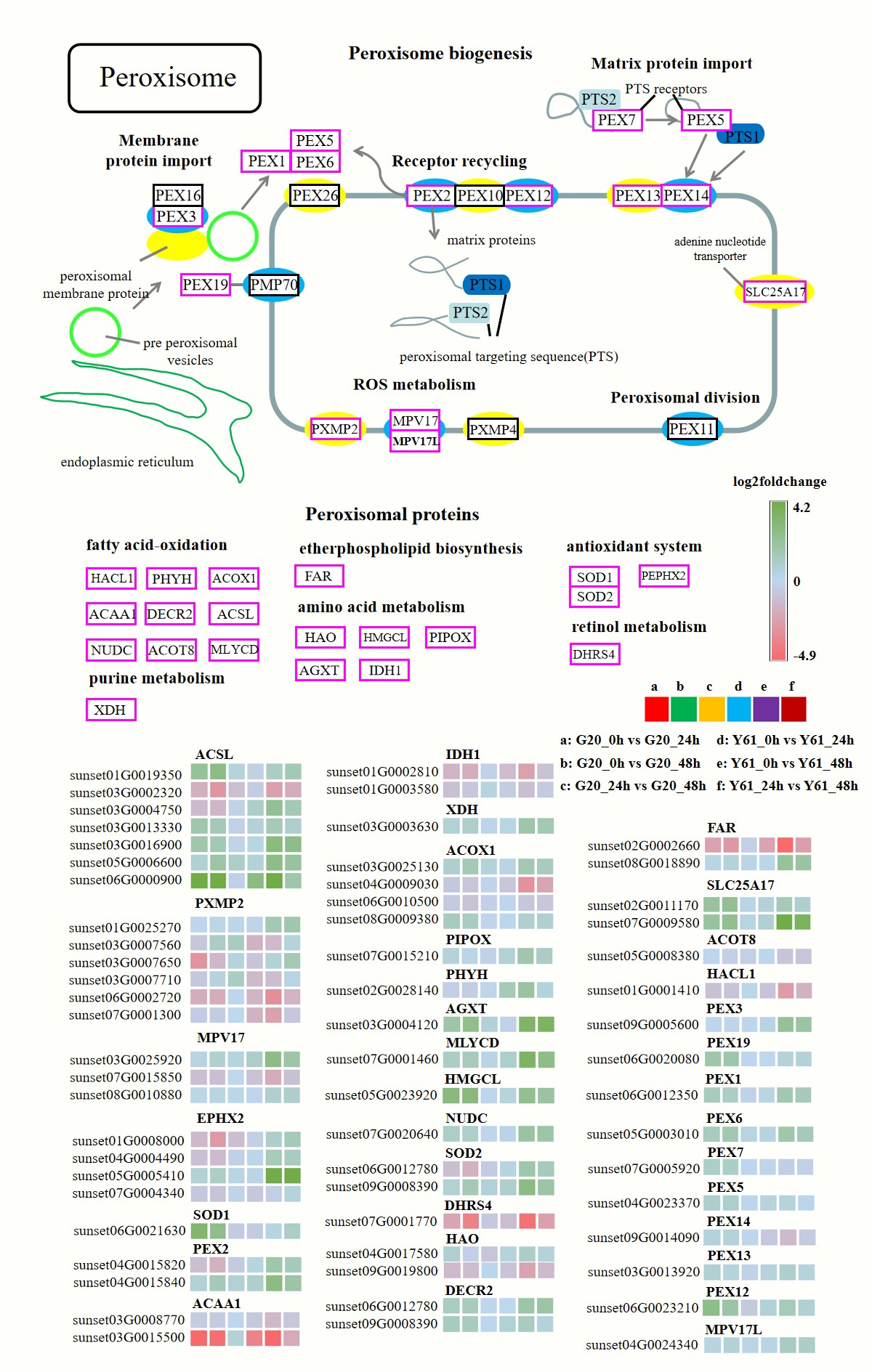

Supplement: Supplementary Figure 1 — Differentially expressed genes in the peroxisome pathway. The fold-change is the ratio of the former group to the latter group. The enzymes invovled are: isocitrate dehydrogenase (IDH1), xanthine dehydrogenase/oxidase (XDH), acyl-CoA oxidase (ACOX1), sarcosine oxidase/L-pipecolate oxidase (PIPOX), phytanoyl-CoA hydroxylase (PHYH), alanine-glyoxylate transaminase/serine-glyoxylate transaminase/serine-pyruvate transaminase (AGXT), malonyl-CoA decarboxylase (MLYCD), hydroxymethylglutaryl-CoA lyase (HMGCL), long-chain acyl-CoA synthetase (ACSL), NAD+ diphosphatase (NUDC), superoxide dismutase, Fe-Mn family (SOD2), superoxide dismutase, Cu-Zn family (SOD1), peroxin-2 (PEX2), acetyl-CoA acyltransferase 1 (ACAA1), soluble epoxide hydrolase/lipid-phosphate phosphatase (EPHX2), dehydrogenase/reductase SDR family member 4 (DHRS4), (S)-2-hydroxy-acid oxidase (HAO), acyl-CoA thioesterase 8 (ACOT8), 2-hydroxyacyl-CoA lyase (HACL1), 2,4-dienoyl-CoA reductase [(3E)-enoyl-CoA-producing], peroxisomal (DECR2), peroxin-3 (PEX3), peroxin-19 (PEX19), peroxin-1 (PEX1), peroxin-6 (PEX6), peroxin-7 (PEX7), peroxin-5 (PEX5), peroxin-12 (PEX12), peroxin-13 (PEX13), peroxin-14 (PEX14), peroxisomal membrane protein 2 (PXMP2), protein Mpv17 (MPV17), Mpv17-like protein (MPV17L), solute carrier family 25 (peroxisomal adenine nucleotide transporter), member 17 (SLC25A17), and alcohol-forming fatty acyl-CoA reductase (FAR). [file Image_1.jpeg]
