# Supplementary material for: Comparative transcriptomics and genomic analyses reveal differential gene expression related to Colletotrichum brevisporum resistance in papaya (Carica papaya L.)
Source: Front Plant Sci. 2022 Dec 23;13:1038598. doi: 10.3389/fpls.2022.1038598 (PMC9816866; doi:10.3389/fpls.2022.1038598)
Supplement: Supplementary Table 1 — Identification results of resistance to anthracnose in two papaya cultivars Y61 and G20. [file Table_1.docx]

**Table S1 Identification results of resistance to anthracnose in two papaya cultivars Y61 and G20**

| Evaluation criterion of resistant degree | The disease index | G20 | Y61 |
| --- | --- | --- | --- |
| High resistance (HR) | 0 |  |  |
| Resistance (R) | 0.1~10.0 |  |  |
| Middle resistance (MR) | 10.1~30.0 | 15.6±2.2 |  |
| Middle susceptible (MS) | 30.1~40.0 |  |  |
| Susceptible (S) | 40.1~50.0 |  |  |
| High susceptible (HS) | >50.1 |  | 60.0±3.3 |

Data shown are means ±SD of three independent biological replicates.
